# On the choice of the phase difference increment in radiofrequency-spoiled gradient-echo magnetic resonance imaging of liquids with consideration of diffusion

**DOI:** 10.1371/journal.pone.0324455

**Published:** 2025-05-30

**Authors:** Jochen Leupold, Matthias Weigel, Sébastien Bär

**Affiliations:** 1 Division of Medical Physics, Department of Diagnostic and Interventional Radiology, University Medical Center Freiburg, Faculty of Medicine, University of Freiburg, Freiburg, Germany; 2 Translational Imaging in Neurology (ThINk) Basel, Department of Biomedical Engineering, Faculty of Medicine, University Hospital Basel and University of Basel, Basel, Switzerland; 3 Department of Neurology, University Hospital Basel, Basel, Switzerland; 4 Research Center for Clinical Neuroimmunology and Neuroscience Basel (RC2NB), University Hospital Basel and University of Basel, Basel, Switzerland; 5 Division of Radiological Physics, Department of Radiology, University Hospital Basel, Basel, Switzerland; PLOS, UNITED KINGDOM OF GREAT BRITAIN AND NORTHERN IRELAND

## Abstract

In magnetic resonance imaging, the radiofrequency-spoiled gradient-echo method aims for fast acquisition of T1-weighted images. The spoiling mechanism is driven by the radiofrequency phase difference increment. In clinical (in-vivo) imaging, the phase difference increments of 50°, 115.4°, 117° and 150° are in standard use. In this work, we examine how accurate these increments guarantee T1-weighting also in free liquids, in particular with different diffusion coefficients. The non-standard phase difference increment 169°, which was shown to improve T1 quantification methods, is considered as well. Signal simulations were performed with the extended phase-graph with diffusion concept; experiments were performed on different liquid phantoms (water with contrast medium, silicone oil). In the simulations, a parameter space consisting of relaxation times, diffusion coefficient, sequence repetition time, flip angle and image resolution was examined. The resulting efficiency of radiofrequency spoiling was quantified by the average deviation of the simulated signal-vs-flipangle curve from the ideal curve. It was found that ideal spoiling is generally better approximated with a phase difference increment of 169° compared to the other examined values. From the four commonly used values, 115.4° is recommended, in particular when the influence of diffusion is low. For clinical in-vivo imaging parameters, all examined values of the phase difference increments offer a good approximation of ideal spoiling as expected. In conclusion, radiofrequency spoiling in free liquids can be improved by using a phase difference increment of 169°.

## Introduction

Radiofrequency(RF)-spoiled gradient-echo sequences, commonly known by acronyms such as FLASH, SPGR or T1-FFE, find wide application in clinical MRI, since they enable T1-weighted imaging and fast 3D volume acquisition [1]. The RF-spoiling mechanism consists of an unbalanced gradient-echo sequence in combination with a quadratically increasing phase of the RF excitation pulses [2–4]. The actual RF phase cycle is determined by the phase difference increment ψ, which is chosen to eliminate the influence of T2 from the recorded voxel signal and therefore to obtain pure T1-weighting (“ideal spoiling”). Several empirical values for ψ are in use; they differ between the implementations of different vendors, e.g., ψ = 50° (Siemens), ψ = 115.4° (GE), ψ = 117° (Bruker) and ψ = 150° (Philips). However, the elimination of T2-contribution to the signal is only approximately fulfilled [5], which was shown to introduce errors to methods that quantify relaxation time T1 or the transmit B1 field [6,7] based on a signal equation without T2 terms. Besides, to verify the extinction of T2 in the signal equation, Yarnykh [6] showed that diffusion cannot be neglected when the effectiveness of RF-spoiling is evaluated. In that study, ψ = 169° is proposed as the best choice for T1-determination in the regime of intermediate-to-strong diffusion influence. The influence of diffusion on the RF-spoiled signal is only rarely considered in the literature; apart from Yarnykh [6] one can find it in Heule et al. [8] and Corbin/Callaghan [9], both papers examine the influence of imperfect spoiling on T1-determination.

In this work, we examine how well different phase difference increments ψ - as used by the different system vendors - approach the ideally spoiled signal if the scope of application is extended from clinical (in-vivo) imaging to imaging of free liquids, such as in studies involving phantoms or contrast media [10,11]. The consequences of different values of ψ on the obtained voxel signal and on the image contrast in 3D GRE acquisitions are analyzed. Experiments were performed on different phantoms with different relaxation times and diffusion coefficients. Numerical simulations were conducted over a wide range of the parameters T1, T2, repetition time TR, flip angle α and isotropic diffusion coefficient D.

### Theory

In a RF-spoiled GRE sequence with RF phase difference increment ψ, the phase *φ*_*n*_ of the *n*-th RF-pulse is (with n ≥ 0) [2,3]


$$\phi_{n}=\sum_{k=0}^{n}k\psi =n(n+1)\psi /2$$ (1)

In combination with the spoiler gradient that sets up a distribution of isochromats with precession angle θ per repetition time TR, the RF-phase cycling according to Eq. 1 leads to a complicated shape of the complex-valued transverse magnetization $M_{n}^{+}(\psi ,\theta$ and $M_{n}^{-}(\psi ,\theta )$ directly after and before the *n*-th RF-pulse, respectively. A pseudo steady-state is obtained for $M_{n}^{+}(\psi ,\theta$ and $M_{n}^{-}(\psi ,\theta )$ [2,4], and the measured amplitude of the voxel signal ${S(\psi )}^{+}$ used for acquisition becomes independent of *n* (i.e., it constitutes a steady state) and can be written as


$${S(\psi )}^{+}=\int_{-\pi }^{\pi }M_{n}^{+}(\psi ,\theta )d\theta \;.$$ (2)

In analogy, the signal that could be measured at the end of the sequence interval is


$${S(\psi )}^{-}=\int_{-\pi }^{\pi }M_{n}^{-}(\psi ,\theta )d\theta .$$ (3)

Note that both $M_{n}^{+}$ and $M_{n}^{-}\;$ depend in general also on T1, T2, TR, flip angle α and diffusion coefficient D (for isotropic diffusion). For better readability, here and in the following, only the parameters that are under concrete consideration are indicated as variables.

**Fig 1:**
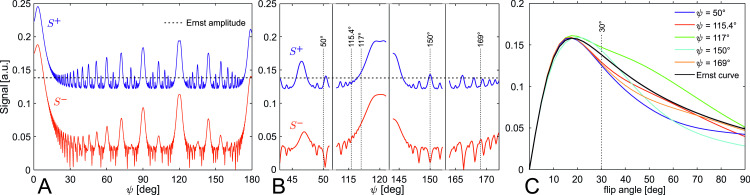
Simulated RF-spoiled signals for T1/TR = T2/TR = 20, α = 30°, no diffusion. (A) Signals *S*^+^ and *S*^-^ in dependency of the RF phase difference increment ψ. (B) Location of the four ψ-values used in practice (ψ = 50°, ψ = 115.4°, ψ = 117°, ψ = 150°) and ψ = 169°. (C) Signal *S*^+^ and the Ernst curve (ideal spoiling) in dependency of the flip angle (α = 30° as used in A and B is indicated).


The ψ-dependency of $S^{+}$ is shown in Fig 1A, with parameters T1/TR = T2/TR = 20, α = 30°, as used in Zur et al. [3] and Duyn [12]. The horizontal line in Fig 1A is the so-called Ernst amplitude

$$S_{\text{Ernst}}^{+}(\frac{TR}{T1},\alpha )=M_{0}\frac{1-e^{-TR/T1}}{1-e^{-TR/T1}\text{cos}\alpha }\text{sin}\alpha ,$$ (4)

which is the amplitude of the voxel signal obtained for $M_{n}^{-}(\psi ,\theta )=0$ that would necessarily lead to ${S(\psi )}^{+}=S_{\text{Ernst}}^{+}$. In Eq. 4, the T2-dependency is removed from the voxel signal and therefore “pure” T1-weighted imaging becomes feasible. This effect is called “ideal spoiling” when Eq. 4 is valid. For TR>>T2, $M_{n}^{-}(\psi ,\theta )=0$ is approximated due to T2-relaxation during TR.

The value $S_{\text{Ernst}}^{+}(TR/T1,\alpha )$ is maximized at the “Ernst angle” $\alpha_{E}$ with


$$\alpha_{E}=\text{arccos}{(e}^{-TR/T1})$$ (5)

Now, the idea of RF-spoiling is to choose the phase difference increment ψ such that ${S(\psi )}^{+}=S_{\text{Ernst}}^{+}$, i.e., ideal spoiling is obtained [3] even without fulfilling TR>>T2. Consequently, candidate values for ψ are those with ${S(\psi )}^{+}\approx S_{\text{Ernst}}^{+}$ independent of T1/TR, T2/TR and α. Such ψ-values are not necessarily the crossing points of the graphs of ${S(\psi )}^{+}\;$and $S_{\text{Ernst}}^{+}$ in Fig 1A, since Fig 1A is simulated for only one parameter set for illustration. Note, the original and simple idea of ideal spoiling with $M_{n}^{-}(\psi ,\theta )=0\;$ is never realized anyway, since these crossing points neither imply $M_{n}^{-}(\psi ,\theta )=0$ nor ${S(\psi )}^{-}=0$ (Eq. 3). Thus, the validity of Eq. 4 when employing RF-spoiling is the result of the pseudo steady-state 3D magnetization vector and originates not from ${S(\psi )}^{-}=0$. When it comes to the choice of a concrete ψ, the approach to ideal spoiling preferably holds for various values of T1/TR, T2/TR and α, i.e., the deviation of ${S(\psi ,\alpha )}^{+}\;$ from the “Ernst curve” $S_{\text{Ernst}}^{+}(\alpha )$ should be small.

Several values for ψ are used in the standard implementations of different MR system vendors: ψ = 50° (Siemens), ψ = 115.4° (GE), ψ = 117° (Bruker) and ψ = 150° (Philips). These values are usually hard coded and are not available as a selectable parameter to the operator. However, each of these values leaves questions open:

First, as shown by simulation in Fig 1B and as noticed above, none of these values indicates a crossing point of ${S(\psi )}^{+}\;$and $S_{\text{Ernst}}^{+}$. Second, the curves ${S(\psi ,\alpha )}^{+}\;$show remarkable deviation from the Ernst curve $S_{\text{Ernst}}^{+}(\alpha )$ in Fig 1C (see also Ganter [5]). Third, we deduce from Fig 1A and 1B that ${S(\psi )}^{-}$ is neither zero for any ψ nor do the crossing points of ${S(\psi )}^{+}\;$and $S_{\text{Ernst}}^{+}$ even correspond to local minima of $\;{S(\psi )}^{-}$ in general, which makes the conception of eliminated transverse magnetization in the sense of $\;{S(\psi )}^{-}=0$ (Eq. 3) at the end of the sequence interval obsolete.

Consequently, RF-spoiling manipulates the 3D magnetization vector dynamics such that the spoiling process is better described as “an attempt to restore the contrast properties of long-TR gradient echo techniques” [4] instead of drawing the picture of *eliminated* transverse magnetization at the end of the sequence interval.

The described circumstances leave the question open according to which criteria the ψ-values for standard sequence implementations have been chosen. Therefore, in this work we examine the differences of the four common ψ-values (ψ = 50°, ψ = 115.4°, ψ = 117°, ψ = 150°) and also ψ = 169° as originally recommended for T1-mapping based on the RF-spoiled GRE signal from Eq. 4 [6].

We consider two criteria for evaluation of the different ψ-values:

1) The Ernst curve (Eq. 4) should closely be matched over a broad range of T1/TR and T2/TR combinations.
2) Not only imaging of human tissue is considered, but also imaging of free liquids that are neither part of the human body nor of other biologic samples (e.g., silicone oil [10,11]).

For this evaluation, isotropic diffusion is assumed, and different values of the diffusion coefficient D are considered as well as varying image resolution leading to varying diffusion weighting. When diffusion is considered, the signal depends not only on the ratios T1/TR and T2/TR as in the common parametrization of the underlying rotation matrices [4], but also on TR [13]. Therefore, variation in TR was also considered for fixed ratios T1/TR and T2/TR.

The analysis was performed on selected phantoms and with MR signal simulations under variation of the parameters T1/TR, T2/TR, TR, α and D.

## Methods

### Phantom experiments

In a first illustrating set of experiments in a 7 cm volume coil, the signal and contrast variation that can be obtained with different ψ-values in RF-spoiled GRE images was demonstrated. A Gadolinium(Gd)-doped water phantom (T1 = 1603 ms, T2 = 613 ms) was imaged together with the silicone oil phantom for ψ ∈{50°,115.4°,117°,150°,169°}. The sequence protocol as described below was used with α = 60°. Images were obtained by Fourier transform of the raw data with MATLAB, i.e., the voxel intensity is linearly mapped to the grey scale. No further windowing was applied.

For systematic analysis, two different liquids were examined and compared with signal simulations:

a) H_2_O doped with CuSO_4_ with T1 = 540 ms, T2 = 340 ms and D = 1.93 ∙ 10^-3^ mm^2^/s;
b) Silicone oil with T1 = 1290 ms, T2 = 399 ms and D = 0.0055 ∙ 10^-3^ mm^2^/s.

Parameters were measured with standard methods [T1 measurement: saturation recovery RARE (vendor name T1map RARE); T2 measurement: multi-spinecho sequence (MSME); D measurement: Stejskal-Tanner spin echo sequence with EPI readout (DTI_EPI)].

Experiments were performed on a 7 Tesla Bruker Biospec 70/20 imaging system, liquids were put into 10 mm NMR tubes that were placed into a 2-channel volume coil with 3.8 cm inner diameter. The tube was centered in the gradient bore with an inner diameter of approx. 12 cm. This setup avoids an impairment of diffusion quantification by gradient field distortions [14].

Imaging experiments were performed with the vendor’s RF-spoiled 3D GRE sequence, modified so that the possibility of varying the RF phase difference increment was added. The imaging parameters for all performed experiments were: TR = 20 ms, TE = 4 ms, FOV 30 mm × 30 mm × 50 mm, matrix 100 × 100 × 64, resolution 0.30 mm × 0.30 mm × 0.78 mm, readout bandwidth 50 kHz, total scan time 2 min 8 s. Spoiler gradients were switched in readout and slice direction with 3 cycles per voxel (spoiling moment of 3 ∙ 2π) and 3.76 cycles per voxel, respectively.

Measurements were conducted from α = 5° up to α = 90° in 5° steps. A region-of-interest (ROI) was evaluated on the central slice. The mean of the ROI was used as signal value. Since the receiver gain was unchanged for different acquisitions and only the flip angle or ψ was varied, the noise contribution remains unchanged. Thus, an explicit SNR (signal-to-noise ratio) calculation was not necessary. Curves ${S(\psi ,\alpha )}^{+}$ were obtained for ψ ∈{50°,115.4°,117°,150°,169°}. Since simulations show that at the Ernst angle α_E_ the curves for different ψ-values have identical values, all curves were normalized to $S_{\text{Ernst}}^{+}\left(TR/T1,\alpha_{E}\right)$, i.e., to the measured values ${S(\psi ,15^{\circ })}^{+}$ and ${S(\psi ,10^{\circ })}^{+}$ for the H_2_O+CuSO_4_ (α_E_ = 15.5°) and silicone oil phantom (α_E_ = 10.1°), respectively. Any possible influence of T2* was neglected, since this would only result in multiplying all considered signals with exp(-TE/T2*) [15] that would cancel by the signal normalization.

The phantom image data and signal values from the ROI evaluation are available for download at https://github.com/leupoldj/psis.

### Quantification of signal deviation from the ideally spoiled signal

In this work, the deviation of the signal curve $S^{+}$ (Eq. 2) from the Ernst curve $S_{\text{Ernst}}^{+}$ (Eq. 4) is quantified by the parameter ε, which is the flip-angle averaged absolute value of the relative deviation (or error) of $S^{+}$ from $S_{\text{Ernst}}^{+}$ in percent. With *N* as the number of flip angles,ε writes to


$$\varepsilon (\psi ,T1,T2,TR,\alpha ,D)=\frac{100\;\%}{N}\sum_{k=1}^{N}\frac{\left|{S(\psi ,T1,T2,TR,\alpha_{k},D)}^{+}-{S(\alpha_{k},TR/T1)}_{\text{Ernst}}^{+}\right|}{\left|{S(\alpha_{k},TR/T1)}_{\text{Ernst}}^{+}\right|}$$ (6)

Consequently, the smaller ε, the better is the spoiling capability of the corresponding ψ.

### Simulations

Signals $S^{+}$ were computed with the extended phase graph with diffusion formalism, according to Weigel et al. [13,16]. The imaging gradient amplitudes and timing parameters of the sequence protocol with imaging parameters from above were used to calculate the influence of diffusion (see S1 Fig). Only gradients in readout direction and the slice spoiler gradient were considered, since other gradients (phase encoding, slice selection) are balanced and their contribution to diffusion weighting can be neglected [17,18]. Three sets of simulations were conducted in MATLAB:

a) Simulation of the measured signal-vs-flipangle curves for the H_2_O+CuSO_4_ phantom and silicone oil phantom.
b) Simulation of signal curves over a range of T1/TR and T2/TR values for ψ ∈{50°,115.4°,117°,150°,169°}. T1/TR and T2/TR were logarithmically varied from log_10_(T1/TR) = -1 to log_10_(T1/TR) = 2.3 in steps of 0.1, corresponding to T1/TR ranging from 0.1 to 200 (the same was done for T2/TR, considering T2 ≤ T1). The influence of TR on fixed T1/TR and T2/TR ratios was investigated by comparing the simulated signal curves for TR = 20 ms and TR = 50 ms. For any signal curve, the parameter ε quantifying deviation from the Ernst curve was calculated according to Eq. 6. A number of *N* = 18 flip angles α were simulated, with α_k_running from 5° to 90° in 5° steps.

The described simulations were performed for four different diffusion coefficients: D = 0 mm^2^/s (no diffusion), D = 0.0055 ∙ 10^-3^ mm^2^/s (silicone oil phantom representing low influence of diffusion), D = 0.8 ∙ 10^-3^ mm^2^/s (representing human brain grey matter [6]) and D = 1.93 ∙ 10^-3^ mm^2^/s (H_2_O+CuSO_4_).

c) For ψ ∈{50°,115.4°,117°,150°,169°}, ε was computed according to Eq. 6, but for different voxel sizes in readout direction. To simulate the effect of different voxel sizes in the calculation, the gradient amplitudes were scaled accordingly. For example, halved voxel size (corresponding to doubled resolution) is realized by doubling both the amplitudes of the gradients in the readout direction and the amplitude of the slice spoiler gradient. Such simulations were performed for both phantoms and – as one example for human tissue - for brain grey matter at 3 Tesla with T1 = 1500 ms, T2 = 100 ms and D = 0.8 ∙ 10^-3^ mm^2^/s [6]. This simulation of the voxel size effect was repeated with TR = 50 ms to see the influence of increased TR in the selected scenarios.

## Results

### Phantom experiments

**Fig 2:**
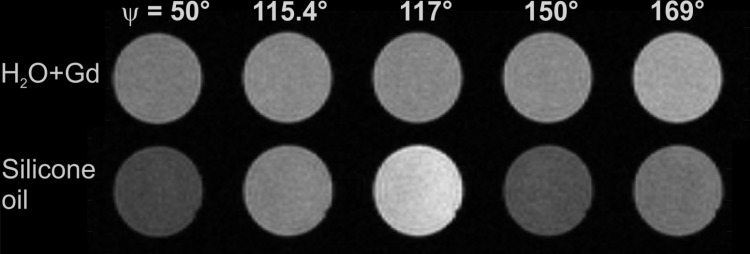
Illustration of contrast varying with ψ. H_2_O+Gd Phantom (top row) and the silicone phantom (bottom row) imaged with different ψ-values (columns) but otherwise identical imaging parameters (TR = 20 ms, α = 60°). Different ψ-values result in different contrast.

Fig 2 illustrates that the contrast between the H_2_O+Gd phantom (top row) and the silicone oil phantom (bottom row) depends on ψ. For the silicone phantom, signal variation is clearly visible. Since the H_2_O+Gd phantom shows only very little intensity variation due to the influence of diffusion on the spoiling process, the contrast between both phantoms varies with ψ. The contrast is even inverted when going from ψ = 117° to ψ = 50° or to ψ = 150°. This means, in such a scenario, different image contrast is obtained on different MRI systems (with the same field strength), despite the same pulse sequence is supposedly used.

**Fig 3:**
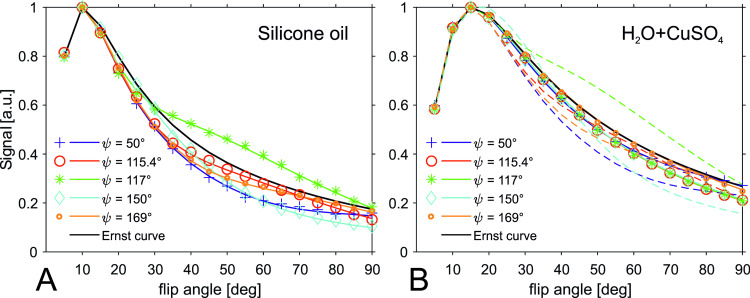
Measurements (markers) and simulations (solid lines) of the RF-spoiled Signal *S*^*+*^. (A) Silicone oil phantom with T1 = 1290 ms, T2 = 399 ms, TR = 20 ms, D = 0.0055 ∙ 10^-3^ mm^2^/s. All curves deviate remarkably from the ideal spoiling case (Ernst curve, black line). (B) H_2_O+CuSO_4_ phantom with T1 = 540 ms, T2 = 340 ms, TR = 20 ms, D = 1.93 ∙ 10^-3^ mm^2^/s. Dashed lines are the simulated curves without diffusion to highlight the impact of diffusion.

Results of the phantom experiments (H_2_O+CuSO_4_ phantom and silicone oil phantom) and corresponding simulations [see a) in Methods – Simulations] are shown in Fig 3. The correspondence of simulations with experimental data provides evidence for the correctness of the simulations. The ε- values (Eq. 6) for the simulated curves in Fig 3A and 3B can be found in Table 1 (TR = 20 ms).

**Table 1:** ε-Values in percent obtained from Eq. 6 for simulations of different substances.

ψ	Without diffusion			With diffusion					
	Zur et al. [3] T1/TR = T2/TR = 20 (Fig 1)	H_2_O+CuSO_4_ no diff.		Silicone oil		Grey matter		H_2_O+CuSO_4_	
		TR = 20 ms	TR = 50 ms	TR = 20 ms	TR = 50 ms	TR = 20 ms	TR = 50 ms	TR = 20 ms	TR = 50 ms
50°	15.2	15.9	6.5	18.3	9.7	5.7	1.4	3.6	1.8
115.4°	5.5	6.3	2.3	9.3	3.2	6.1	3.7	7.7	4.3
117°	14.6	13.8	5.7	13.8	8.6	5.6	3.7	7.6	4.3
150°	17.9	18.0	13.1	20.0	16.3	8.5	2.4	7.2	3.3
169°	6.1	6.9	1.9	9.6	3.3	1.5	0.8	0.9	2.6

The “with diffusion” values in the table are based on the imaging protocol as used for the experiment, i.e., voxel size = 300 μm in readout direction.

Fig 3A shows the result obtained with the silicone oil phantom. Curves for all ψ-values show deviation from the Ernst curve, with ψ = 115.4° matching best in this example. In Fig 3B, the influence of diffusion is demonstrated by the curves (solid lines and markers) showing the RF-spoiled signal with different ψ-values for the H_2_O+CuSO_4_ phantom. All curves come closer to the Ernst curve than in the simulated artificial case without diffusion (dashed lines – shown to illustrate the impact of diffusion). The best match to the Ernst curve is obtained with ψ = 169° (orange solid curve and markers). Fig 3 also illustrates that the deviation from the Ernst curve for a certain (rather high) flip angle can be higher than it is suggested by ε, which averages over the flip angle (Eq. 6). On the other hand, for a certain flip angle α the best match might be obtained with a ψ-value that differs from the ψ-value with lowest ε. For example, ψ = 50° exhibits the second highest deviation ε for the silicone oil phantom (Table 1) but shows good match to the Ernst curve at α = 90°(Fig 3A). 

The results from Fig 3 confirm that variation of the RF phase difference increment ψ can have practical consequences on the signal and image contrast.

### Simulations

The ε-values (Eq. 6) obtained for the curves in Fig 1C are 15.2%, 5.5%, 14.6%, 17.9% and 6.1% for ψ-values of 50°, 115.4°, 117°, 150° and 169°, respectively (Table 1). These ε-values were determined to illustrate the influence of diffusion on parameters (T1 = T2 = 20TR) regularly used in literature [3,4,7,12].

**Fig 4:**
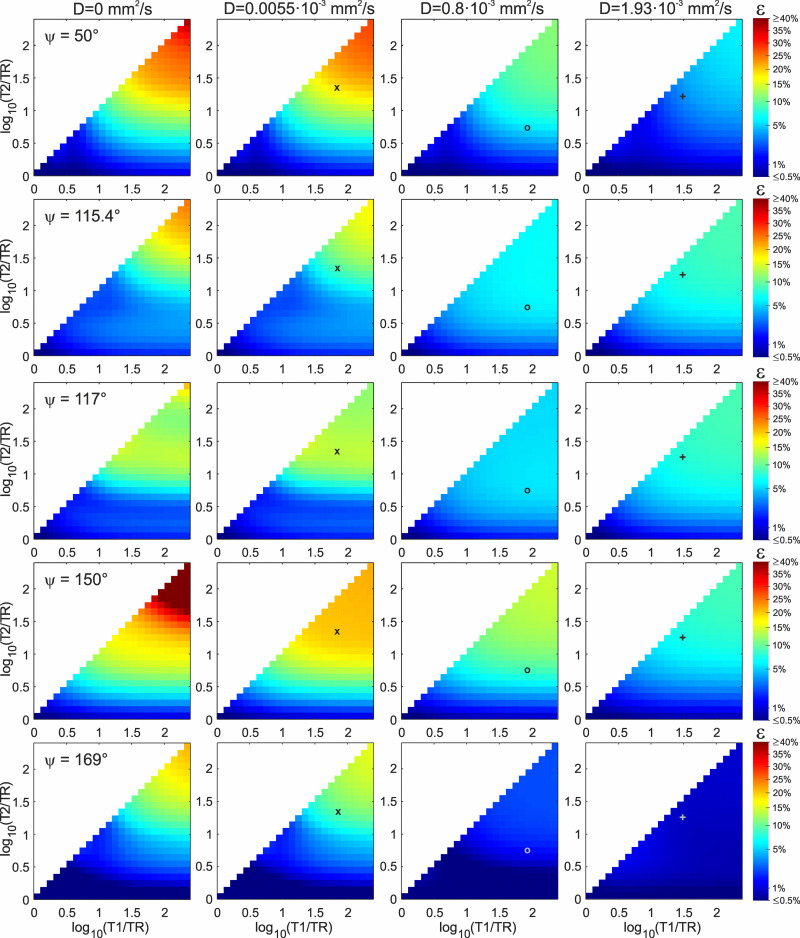
ε-Values according to Eq. 6. The parameter ε quantifies the deviation of the RF-spoiled signal *S*^+^(α) from ideal spoiling, here for variation of T1/TR and T2/TR (TR = 20 ms) and the selected values for ψ (rows) and diffusion coefficient D (columns). The colors represent log_10_(ε). Note also the logarithmic scaling of the colorbar. Relaxation times and diffusion coefficient for the silicone oil phantom (marker **x**, representing the curves in Fig 3A), grey matter at 3 Tesla (marker **o**) and the H_2_O+CuSO_4_ phantom (marker **+** , representing the curves in Fig 3B) are indicated.

**Fig 5:**
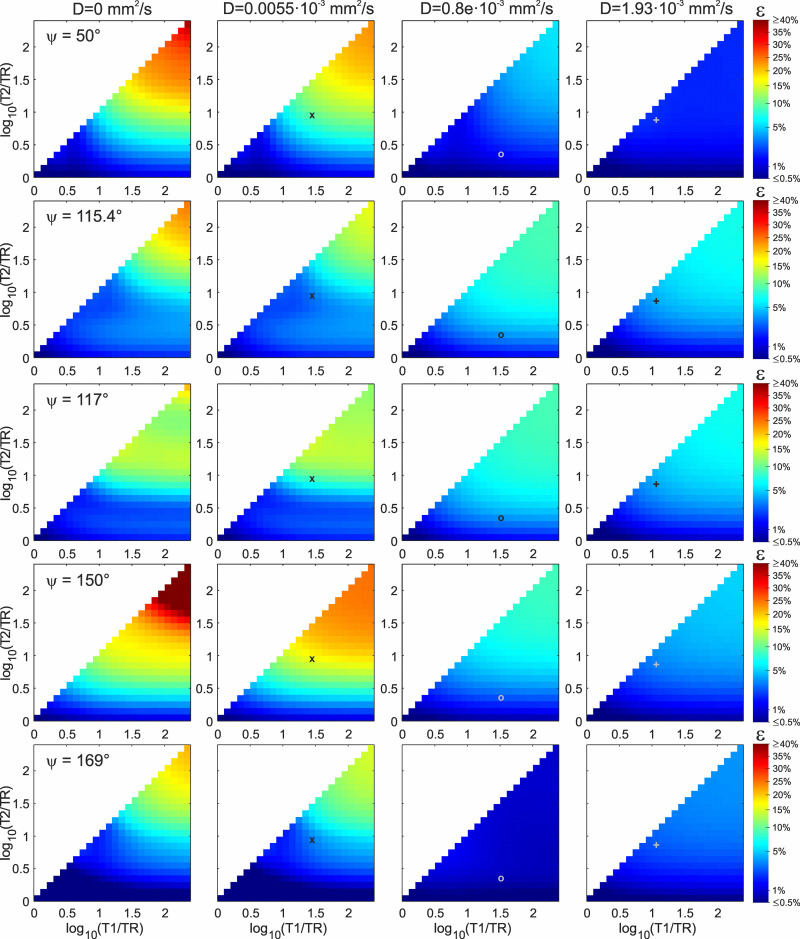
ε-Values as for **Fig 4**. Here with TR = 50 ms.

Simulation results for ε-values in dependency of T1/TR and T2/TR for ψ ∈{50°,115.4°,117°,150°,169°} and D ∈ {0mm^2^/s,0.0055 ∙ 10^-3^mm^2^/s,0.8 ∙ 10^-3^mm^2^/s,1.93 ∙ 10^-3^mm^2^/s} are shown in Figs 4 and 5, for TR = 20ms and TR = 50ms, respectively [see b) in Methods – Simulations]. The ε-values are logarithmically scaled. First of all, it can be concluded that ε-values in the case of T2 < TR (i.e., log_10_(T2/TR)<0) become very small (<1%) as expected and the match to the Ernst curve can be regarded ideal in this case. Therefore, only data with T2 ≥ TR is displayed in Figs 4 and 5.

For the substances corresponding to the diffusion coefficients used for the simulations in Figs 4 and 5 (silicone oil: D = 0.0055 ∙ 10^-3^ mm^2^/s; grey matter: D = 0.8 ∙ 10^-3^ mm^2^/s; H_2_O+CuSO_4_: D = 1.93 ∙ 10^-3^ mm^2^/s), the ε-values are listed in Table 1, for both TR = 20 ms and TR = 50 ms. The maximal ε-value (i.e., maximal mismatch to the Ernst curve) shown in Figs 4 and 5 is 130% for T1/TR = T2/TR = 200 and ψ = 150°, TR = 20 ms. This extreme case vanishes very quickly with the influence of diffusion. Even for the low value of D = 0.0055 ∙ 10^-3^ mm^2^/s, the matching to the Ernst curve improves dramatically. Apart from that, maximal obtained ε-values are approx. 20–30% (orange color in Figs 4 and 5). As expected, with increasing influence of diffusion (higher D or longer TR), ε-values go down and the matching to the Ernst curve improves. However, it is important to note that this behavior represents only a general trend and it can be violated in some cases. For example, ε-values increase despite augmented influence of diffusion in the regime around log_10_(T1/TR) ≈ log_10_(T2/TR) ≈ 1 for ψ = 115.4° and increased D (in Figs 4 and 5), or when comparing TR = 20 ms (Fig 4) with TR = 50 ms (Fig 5) for D = 1.93 ∙ 10^-3^ mm^2^/s and ψ = 169°.

In Figs 4 and 5, ψ = 115.4°, ψ = 117° and ψ = 169° exhibit relatively low ε-values (no orange areas for D > 0mm^2^/s) in comparison to ψ = 50° and ψ = 150°. For medium to high D, ψ = 169° shows clearly the lowest ε-values and offers best spoiling conditions. This means, ψ = 169° is broadly applicable without the danger of falling into a severe mismatch to the Ernst curve.

**Fig 6:**
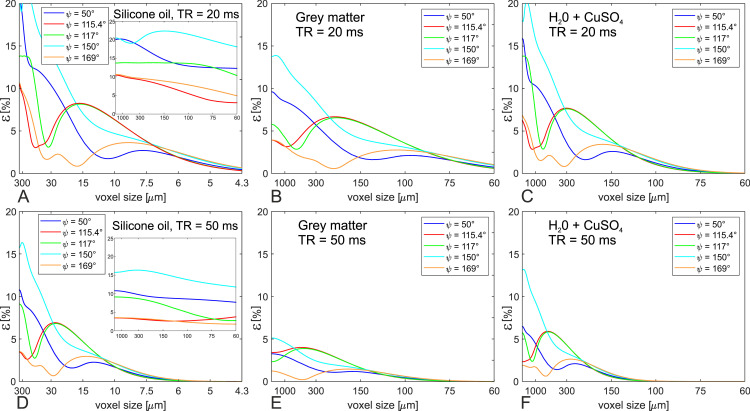
Numerical simulation of spoiling quality (ε-values) in dependence of image resolution and repetition time TR. The voxel size on the x-axis label refers to readout direction. Top row: TR = 20 ms, bottom row: TR = 50 ms. (A)+(D): silicone oil phantom, (B)+(E): grey matter at 3T, (C)+(F): H_2_O+CuSO_4_ phantom.

Consequences of increased diffusion weighting for higher spatial resolution (smaller voxel size) are visible in Fig 6 (see c) in Methods – Simulations]. In comparison with TR = 20 ms (top row), ε-values for the three examined substances scale generally down for TR = 50 ms (bottom row) due to lower T2/TR and increased influence of diffusion. With increased resolution, ε-values approach the limit ε → 0%, although not monotonically, see, e.g., the curves in Fig 6 for ψ = 115.4° (red), ψ = 117° (green) or ψ = 169° (orange). Again, ψ = 169° shows relatively low ε-values over the whole range of voxel sizes. However, for larger voxel sizes and shorter TR (here TR = 20ms), ψ = 115.4° offers the lowest ε-values. For medium to small voxel sizes, ψ = 50° performs best (blue curve in Fig 6).

## Discussion

In the present work, the signal response vs. the excitation flip angle of RF-spoiled GRE sequences was examined for five RF phase difference increments ψ (50°, 115.4°, 117°, 150°, 169°) and possible deviations from the ideal spoiling scenario were quantified. Since the pseudo-steady-state of magnetization resulting from the RF-spoiling mechanism is very difficult to handle analytically [4] and also looks quite chaotic to the human eye [19], it does not come as a surprise that not one single, “global” RF phase difference increment could be found as the best match to the Ernst curve (representing ideal spoiling conditions) for all possible parameter combinations of T1, T2, TR, flip angle, isotropic diffusion coefficient and image resolution. For certain parameter settings, different contrast can be observed also for the MR system vendors’ different implementations (i.e., different ψ-values) of RF-spoiling (Fig 2). This leads to the question of how well the commonly used ψ-values match the Ernst curve in different parameter regimes, since ψ-dependent contrast variation can be observed, in particular for low-diffusion substances.

To approach this goal, the parameter ε was introduced that quantifies the average deviation (per flip angle) from the Ernst curve in percent. Lower ε-values indicate a better matching of the measured “real” signal values to the Ernst curve than higher ε-values. Since the deviation is approximately zero for flip angles lower than the Ernst angle α_E_ (Figs 1C and 2), the deviation at flip angles α > α_E_ (Eq. 5) can be higher than ε.

A certain ψ can be regarded as a good choice if ε is kept as low as possible over the whole parameter space, and no regimes with high ε-values exist. In that sense, ψ = 115.4° and ψ = 169° could be shown to be superior to ψ = 50°, ψ = 117° and ψ = 150° (Figs 4–6). Since diffusion contributes more to the spoiling effect for ψ = 169° than for ψ = 115.4° (see Figs 4 and 5), we recommend to use ψ = 169° in RF-spoiled GRE protocols. However, the other examined phase difference increments have their peculiarities. The value ψ = 117° was suggested by Zur et al. [3] as the location of a crossing point of ${S(\psi )}^{+}\;$ and $S_{\text{Ernst}}^{+}$, however, as pointed out by Duyn [12], Zur and colleagues looked at the absorption part of the Signal $S^{+}\;$(see Fig 7 in Ref. [3]) and not at the magnitude that is used to describe signal and contrast in GRE images. Had Zur et al. considered the magnitude signal instead, they would probably have suggested ψ = 116° or ψ = 115° and the “famous” 117° would never have entered literature. In that sense, ψ = 115.4° can be regarded as a corrected value to the original ψ = 117°. This is in accordance to our results (Figs 4–6), since ψ = 115.4° mostly exhibits lower ε-values than ψ = 117°, in particular when influence of diffusion is low (second column in Figs 4 and 5, large voxel sizes in Fig 6).

The increment ψ = 50° demonstrates to be the best choice at intermediate to small voxel size in Fig 6 (blue curves) for short repetition time (here TR = 20 ms), but the performance at lower resolution is worse in comparison to ψ = 115.4° and ψ = 169°.

For ψ = 150°, there was always an other ψ-value yielding a lower ε than the ε obtained with ψ = 150°. In particular, for any examined parameter combination, ψ = 169° matches the Ernst curve at least as good as ψ = 150°, mostly better. These findings are in contradiction to the statement “For optimal RF spoiling over a wide range of T1, T2, and TR, a phase difference increment of […] 150° is applied” from Treier et al. [20]. Please note in this respect that neither an analysis of such a parameter space was performed there, nor does the reference [3] provided with that sentence does mention a phase difference increment of 150°.

In clinical (in-vivo) imaging, diffusion is of significant influence and low flip angles in the proximity of the Ernst angle are usually applied. With such parameters, all examined ψ-values (and many others) approximate the ideal spoiling scenario very well and “pure” T1-contrast is obtained. This means, in practice the choice of better increments to obtain “pure” T1-contrast can be beneficial when it comes to imaging with flip angles higher than the Ernst angle (see Fig 3). In particular, the ideal spoiling scenario can be severely violated when diffusion effects are low, for instance in phantoms that are designed to suppress diffusion [21]. This can be seen very prominently for ψ = 50° or ψ = 150° in Fig 4. This means, since the readout direction contributes most to the imaging sequence’s induced inherent diffusion weighting, the RF-spoiled GRE signal can become dependent on gradient orientation when diffusion is highly anisotropic, such as in capillary phantoms for signal model evaluation [22,23]. Consequently, the choice of the phase difference increment is not only crucial when a model based on ideal spoiling is used to determine T1 or B1 [6–9], but can also have considerable impact on image contrast (see Fig 2). The deviation from ideal spoiling can be prominent in particular at higher flip angles (see Fig 3) and rather short TR. Consequently, for experiments on ultra high-field human scanners, such protocols may not run due to SAR (specific absorption rate) restrictions [24]. SAR is not an issue on animal scanners or spectrometers with (micro)imaging capability, though.

The influence of diffusion is generally determined by the diffusion tensor, but also by the effective b-value of the sequence, i.e., gradient amplitudes and timing [13,16]. In terms of sequence parameters, this means both higher resolution and longer TR modify the sequence’s inherent diffusion weighting. Although there is a trend of improved approximation of ideal spoiling with longer TR and/or increased resolution, spoiling can also get worse in some parameter regimes when resolution or TR is increased (Fig 6). Besides, the moments of the spoiler gradients are of importance as well. In terms of approximation to ideal spoiling conditions, the effect of a higher moment of a spoiler gradient is qualitatively comparable to an increase in image resolution. Note that the sequence protocol used in our study comes with a voxel size of 300 μm and rather high spoiler gradients with spoiling moments above 6π, which is beyond the commonly recommended moment of 2π [19]. Consequently, this protocol operates under substantial influence of diffusion but nevertheless still leads to considerable deviations from the Ernst curve (Fig 3). Hence, low influence of diffusion and subsequent violation of ideal spoiling is easily obtained in practical scenarios with lower resolution and/or lower spoiling moments, and the deviation from the Ernst curve becomes even more severe.

Please note our evaluation refers to the steady-state that is obtained for the signal *S*^+^ (Eq. 2); however, the vendors might also have taken the signal behavior during the approach to steady state into account for their choice of the phase difference increment ψ [25].

In our study, we have examined more than the five discussed ψ-values. If good spoiling over the whole examined parameter space is intended, no ψ-value could be found that showed better spoiling capability than ψ = 169°. The interested reader might verify this by means of the provided simulation code (https://github.com/leupoldj/psis). This is in accordance with Yarnykh [6] who suggested ψ = 169° as the increment of choice for Ernst-curve-based T1-determination in presence of intermediate to strong diffusion influence. For narrow regions in the parameter space and selected substances, increments with better spoiling capabilities can be found. For instance, ψ = 40.5° showed very good spoiling at short TR and when the influence of diffusion is low (see S2 Fig), such as for the silicone oil phantom at short TR = 20 ms and voxels larger than 200 µm.

## Conclusion

It could be shown that in RF-spoiled gradient-echo sequences the RF phase difference increments ψ = 169° and ψ = 115.4° offer better approximation to ideal spoiling over a wide range of parameters T1/TR, T2/TR, flip angle and diffusion coefficient in comparison with the other popular and commonly used values of ψ = 50°, ψ = 117° and ψ = 150°. When considering a large parameter space and different imaging scenarios such as imaging of free liquids, the RF phase difference increment ψ = 169° is the most recommendable choice as a fixed parameter in RF-spoiled GRE protocols.

## Supporting information

S1 Fig Gradient amplitudes and timing of the sequence protocol. Amplitudes (red) are in mT/m, durations (black) in ms.
(TIF)

S2 Fig ψ = 40.5°. (A) color-coded ε-values as in Fig 4 (TR = 20 ms, top row) and Fig 5 (TR = 50 ms, bottom row). Note the same colorbar is used as in Figs 4 and 5 to allow visual comparison. ψ = 40.5° seems particularly attractive for short TR, low diffusion coefficients and high T2/TR, which are the conditions for which the other ψ-values show diminished spoiling capability (c.f. Figs 4 and 5). (B) Resolution-dependency for the discussed substances, with ψ = 40.5°, ψ = 115.4° and ψ = 169°. Again, ψ = 40.5° is promising for low D and rather large voxels (see the panel for silicone oil with TR = 20 ms), c.f. Fig 6.
(TIF)
