# Supplementary figures and images for: On the choice of the phase difference increment in radiofrequency-spoiled gradient-echo magnetic resonance imaging of liquids with consideration of diffusion
Source: PLoS One. 2025 May 30;20(5):e0324455. doi: 10.1371/journal.pone.0324455 (PMC12124847; doi:10.1371/journal.pone.0324455)

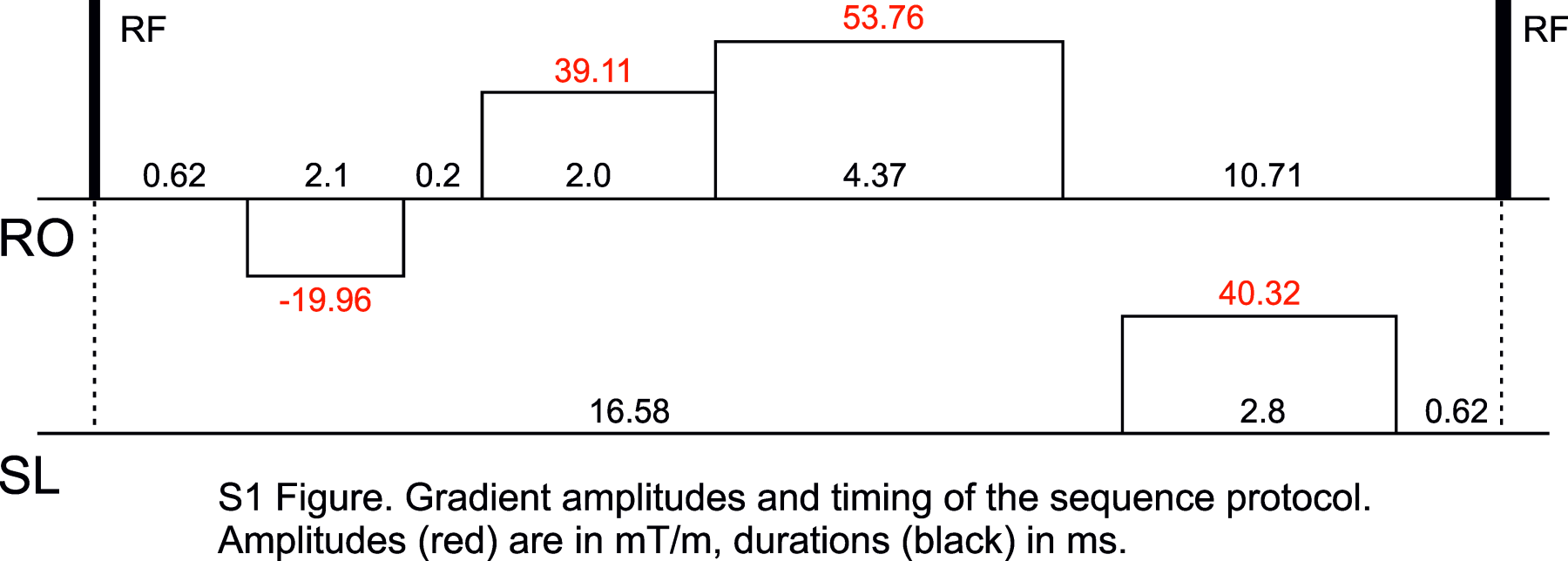

Supplement: S1 Fig — Amplitudes (red) are in mT/m, durations (black) in ms. (TIF) [file pone.0324455.s001.tif]

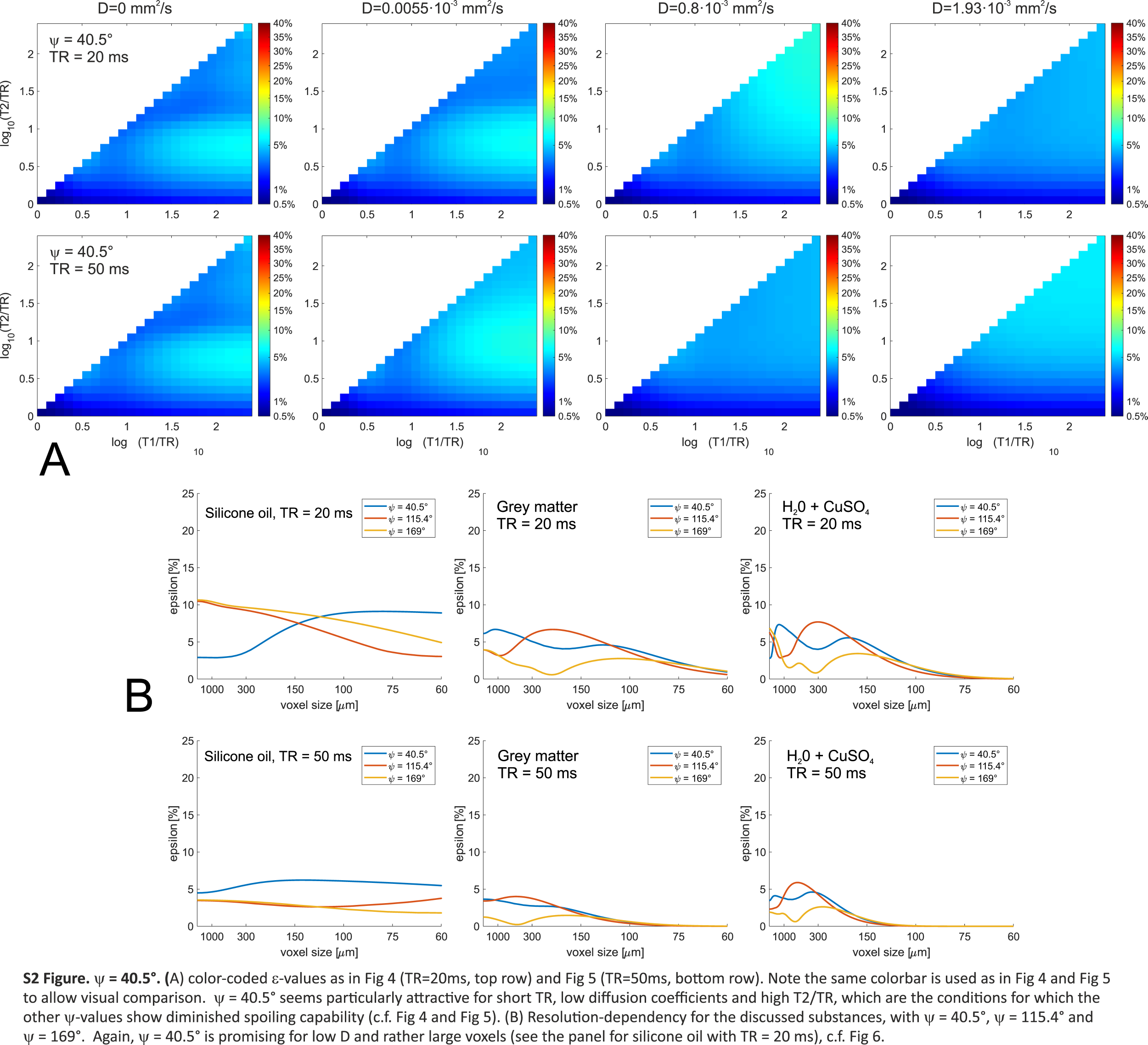

Supplement: S2 Fig — (A) color-coded ε-values as in Fig 4 (TR = 20 ms, top row) and Fig 5 (TR = 50 ms, bottom row). Note the same colorbar is used as in Figs 4 and 5 to allow visual comparison. ψ = 40.5° seems particularly attractive for short TR, low diffusion coefficients and high T2/TR, which are the conditions for which the other ψ-values show diminished spoiling capability (c.f. Figs 4 and 5). (B) Resolution-dependency for the discussed substances, with ψ = 40.5°, ψ = 115.4° and ψ = 169°. Again, ψ = 40.5° is promising for low D and rather large voxels (see the panel for silicone oil with TR = 20 ms), c.f. Fig 6. (TIF) [file pone.0324455.s002.tif]
